# A new effective treatment for dyslexia based on dorsal visual stream neuromodulation

**DOI:** 10.1186/s11689-026-09675-3

**Published:** 2026-01-31

**Authors:** Giuseppe Di Dona, Denisa Adina Zamfira, Francesco De Benedetto, Chiara Turri, Camilla Venturini, Lisa Venniro, Daniela Perani, Luca Ronconi

**Affiliations:** 1 School of Psychology, Vita-Salute San Raffaele University, Via Olgettina 58, 20132 Milano, (MI) Italy; 2 Division of Neuroscience, IRCCS San Raffaele Scientific Institute, Via Olgettina 60, 20132 Milano, (MI) Italy; 3 Department of Psychology and Cognitive Science, University of Trento, Corso Bettini 84, 38068 Rovereto, (TN) Italy

**Keywords:** Non-invasive brain stimulation, tACS, Beta oscillations, Dorsal visual stream, Magnocellular system, Reading, Reading acceleration, Learning disabilities

## Abstract

Developmental dyslexia (DD) affects approximately 10% of individuals, impairing reading ability thus limiting professional fulfilment and psychological wellbeing. DD is associated with both phonological and visual deficits, and the latter are attributed to dysfunctions of the magnocellular-dorsal visual stream, which has a critical role in planning saccades and supporting the extraction of letters/words identity. Currently, there are no effective treatments to restore the brain networks underlying visuospatial analysis and oculomotor efficiency. Adults with DD were enrolled in a randomised clinical trial introducing a non-invasive neuromodulation protocol specifically designed to enhance dorsal stream functionality. We used bi-focal beta-band transcranial alternating current stimulation (tACS) in parietal areas, due to the recognized role of beta oscillation in the dorsal stream functionality. Coupled with a 12-session visuoattentional training, such protocol induced improvements in reading speed, oculomotor control, and visual motion perception. Additionally, beta-tACS led to long-term enhancement of working memory. These outcomes were exclusive or superior to those obtained with a placebo/sham neuromodulation, and were accompanied by plastic changes in the stimulated brain networks. Overall, our findings show the efficacy of multisession tACS in improving core visual, oculomotor and cognitive deficits associated with reading disorders.

**Supplementary Information:**

The online version contains supplementary material available at 10.1186/s11689-026-09675-3.

## Significance statement

The present study introduced an innovative approach to improve reading abilities in adults with developmental dyslexia (DD), combining visual-attentional training with transcranial alternating current stimulation (tACS) at beta (18 Hz) frequency targeting the parietal cortex. This intervention significantly improved reading speed, optimised oculomotor control, and improved working memory. Such benefits were superior to the ones obtained with visuoattentional training alone. These cognitive and oculomotor improvements were accompanied by plastic changes in neurophysiological activity. These results provide crucial insights into DD's neurobiological underpinnings and highlight a novel, effective, and safe approach to treating DD-related visual deficits, offering promising therapeutic potential also for other neurodevelopmental disorders.

## Introduction

Developmental dyslexia (DD) is a multifactorial neurodevelopmental disorder characterised by impaired reading abilities [1] where reading deficits depends on impairments affecting different cognitive domains (e.g., vision, attention, auditory processing, memory) [2–7]. Despite its multifactorial nature, currently available remediation programs focus mainly on improving phonological skills [8–10], reestablishing the functionality of the language network of the left hemisphere [11, 12]. Nonetheless, a consistent amount of evidence shows robust links between visuoattentional/oculomotor impairments and dysfunctions of neural activity along the magnocellular-dorsal (hereafter, dorsal) visual stream in the DD population [4, 13–19].

The dorsal stream is the fastest visual sub-pathway responsible for locating and timing the order of visual events in the visual field to be further processed in the slower Parvocellular Ventral (P–V) stream, which instead is involved in object identification [20, 21]. The dorsal system is responsible for localising the boundaries of individual letters/words, providing essential spatiotemporal information for subsequent identification [19, 22, 23].

A deficiency along the pathway from the Lateral Geniculate Nucleus to the Superior Parietal Area – the main cortical projection of the dorsal stream – can impair dorsal-to-ventral communication, resulting in a poorer ability to correctly integrate/segregate letters/words in the visual field [13, 24], and in excessive visual crowding [25–27]. Importantly, oculomotor control – which also depends on the dorsal stream input– is severely limited in individuals with DD, who struggle in planning/executing optimal saccades [28, 29].

Recent evidence has linked many functions of the dorsal stream to the beta-band (15–25 Hz) oscillatory activity of the parietal cortex and the fronto-parietal network, including visuospatial attention, spatiotemporal integration/segregation, and crowded letters discrimination [30–39], for a review see [40], as well as saccades preparation/execution [41, 42]. Furthermore, applying beta-band transcranial alternating current stimulation (tACS) to the parietal cortex improves the perception of crowded letters, providing a causal link between beta oscillations and reading-related visual functions [30, 31]. Finally, anomalies in endogenous beta-band activity over occipito-parietal cortices and their link with reading performance have recently been reported in DD [43]. Overall, these results frame beta oscillations as the core rhythm of the parietal areas that is essential for reading, paving the way for the use of neuromodulation for ameliorating visuospatial deficits in DD [23].

In the present study we investigated for the first time a multi-focal neuromodulation protocol where beta-band tACS is applied over superior parietal areas and coupled with a multisession visuoattentional reading training to ameliorate reading and reading-related core deficits of DD. Prolonged beta-band stimulation of the parietal areas was expected to enhance visuospatial analysis of text and oculomotor control, but also other core functions underlying reading (i.e., working memory, visual perception, lexical access), through neuroplastic mechanisms resulting in improved reading efficiency. Results would provide clear evidence of the potential of tACS to induce plastic changes in the brain following repeated application, which is nearly absent in the panorama of neurodevelopmental disorders. Secondly, it may provide a causal link between the functionality of parietal areas and reading deficits. This would bring conclusive evidence of the benefit of tACS in restoring specific neural circuits underlying reading, confirming that visuospatial processes are a necessary target for effective treatment of DD.

## Methods

### Participants & study design

Sample size was estimated based on a priori power analysis using GPower. The employed behavioural training procedure (Breznitz et al.) [44], resulting in a 37% reading speed gain across multiple sessions, would only require 3 participants to be reproduced in the absence of beta-tACS. Assuming that beta-tACS would have induced at least a 5% difference in reading speed gain as compared to the sham/placebo group, to reach a power of 0.8, two groups of *N* = 13 each would be required.

Screening for eligibility was carried out for 60 adult volunteers diagnosed with DD who contacted us with an interest in taking part in the clinical trial. To be eligible for the inclusion, individuals had to be aged between 18 and 35 years old, had a certified diagnosis of Developmental Dyslexia made by a qualified clinician prior to the enrollment in the study (diagnoses were verified by two qualified psychologists, C.T. and F.D.B), had no other comorbid neuropsychiatric conditions (i.e., ADHD, autism or schizophrenia spectrum disorders, and mood disorders) or neurological disorders (i.e., epilepsy, migraine and chronic headache disorders, traumatic brain injury, stroke and transient ischemic attacks). Out of the initial sample, 30 adult Italian participants with a confirmed diagnosis of Developmental Dyslexia, normal or corrected-to-normal vision, and normal hearing were recruited (See Figure S1). All participants met the criteria for the application of tACS [45] and decided to take part in the study without any remuneration. The trial was registered in the ClinicalTrials.gov Protocol Registration and Results System (ID: NCT05583136). The study protocol was approved by the Ethical Committee of San Raffaele Hospital and performed in accordance with the Helsinki Declaration of Human Studies and all participants signed the informed consent prior to participating in the study. After recruitment, participants were randomly assigned to one of the 2 treatment arms using a randomly generated allocation list: Vis-tACS (intervention group) or Vis-Sham (control group). The Vis-tACS group performed 12 training sessions, on separate days over 1 month, of a visuoattentional reading training (Breznitz et al.) [44], while receiving bifocal beta-tACS at 18 Hz targeting bilateral parietal areas. The control group (Vis-Sham) performed the same 12 training sessions but with a sham stimulation. Each training session lasted ~ 1.30 h and each participant performed 2.67 ± 0.8 sessions per week.

Both groups underwent a pre-training (T0) and post-training (T1) neuropsychological assessment of reading efficiency and cognitive functioning, as well as an assessment of EEG/ERPs dynamics of visual perception and lexical processing. In particular, a Coherent Dot Motion task (CDM) and a Lexical Decision task (LD) were introduced to assess magnocellular-dorsal functionality and lexical access, respectively. The CDM task probed visual motion perception abilities which heavily depend on the activity of the dorsal stream [46, 47], such abilities not only are impaired in individuals with DD [48], but also predict future reading development in children with DD and improve together with reading skills following MD-targeted training [13]. The LD task probed lexical access as well as orthographic processing [49, 50], which are consistently hindered in individuals with DD [49, 51, 52]. The outcomes of these two tasks allowed to monitor training-induced and tACS-induced improvements of magnocellular functionality, lexical and orthographic processing at a neurophysiological level. Testing sessions at T0 and T1 included both neuropsychological and EEG/ERP measurements and lasted ~ 2 h, while testing sessions at T2 and T3 included only the neuropsychological assessment and lasted ~ 30 min.

**Fig. 1:**
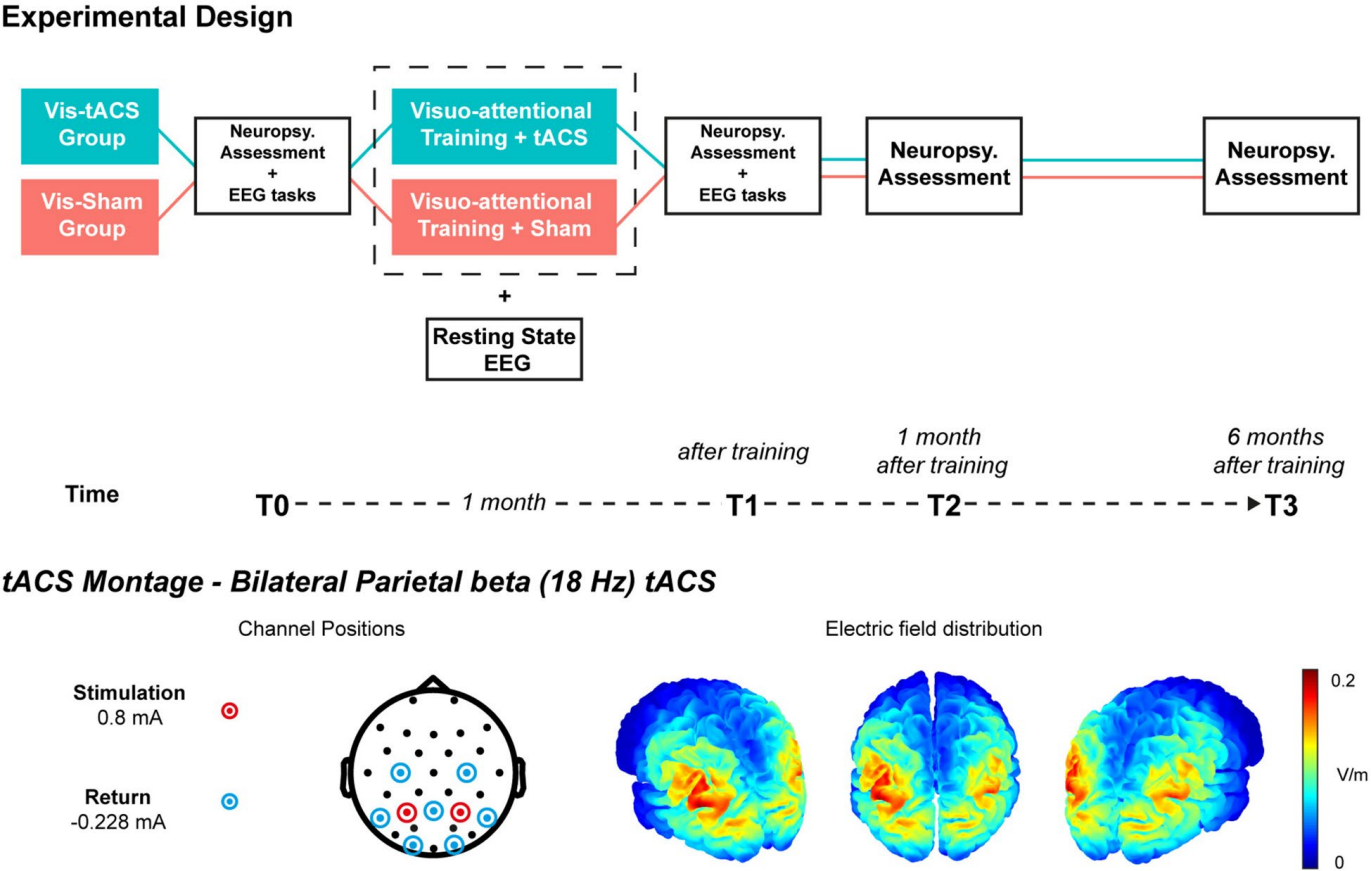
Experimental Design and tACS Montage. The first row shows the experimental design of the study depicting the testing and training procedures over time for each group. The second row shows the tACS montage employed for the Vis-tACS group depicting the position of the stimulation and return electrodes as well as the simulated electric field distribution of tACS

The neuropsychological assessment was repeated after ~ 1 month (T2) and ~ 6 months (T3) after the training (Fig. 1). In addition, computerised reading was recorded in combination with eye-tracker measurements to monitor expected modifications of oculomotor control during reading over the 12 training sessions. Finally, resting state EEG (RS-EEG) data were collected before and after each training session to measure beta oscillatory activity in relevant cortical areas. Upon completion of the study (T3), each participant was requested to indicate which group they believed they had been allocated to (i.e., active or placebo/sham). Subsequently, a comprehensive debriefing session was conducted, during which the true nature and purpose of the study were fully disclosed. This procedure was designed to guarantee that participants had a comprehensive understanding of the study's objectives and to address any potential misconceptions they may have had regarding their participation and group assignment. 3 participants dropped out before the conclusion of the training programme (T1). Thus, the final sample included 27 participants (13 F, Mean Age = 22.44, Age range = 18–32). Participants receiving active (Vis-tACS group) and sham beta-tACS (Vis-Sham group) did not differ in baseline (T0 assessment) reading speed and accuracy evaluated using standardised tests (text, words, pseudowords reading) and had comparable levels of non-verbal intelligence measured with at Raven's Advanced Progressive Matrices (APM; See Table 1). 1 participant was lost at 6 months follow-up (T3; See Figure S1).

**Table 1:** Descriptive statistics of all participants at T0 (excluding drop-outs from T1 onwards) of their age and performance at each cognitive test and independent sample t-tests' results (*p*-values). All measures are reported in z-scores except for Raven’s APM (raw values)

Measure	Vis-tACS Group (*N* = 14) Mean (SD)	Vis-Sham Group (*N* = 13) Mean (SD)	Vis-tACS vs Vis-Sham (p-value)
Age	23.1 (4.43)	21.8 (2.62)	.35
Raven’s APM	49.1 (6.59)	49.7 (5.27)	.81
Word Reading Speed	−2.25 (0.76)	−1.96 (0.90)	.37
Pseudowords reading speed	−2.07 (0.79)	−1.92 (0.64)	.60
Text Reading Time	−3.51 (2.56)	−3.98 (2.72)	.64
Words Reading Errors	−1.33 (1.79)	−2.04 (2.94)	.45
Pseudowords Reading Errors	−1.88 (1.96)	−1.48 (2.09)	.61
Text Reading Errors	−3.21 (2.47)	−2.61 (2.22)	.51

### Procedures

#### Neuropsychological testing

Participants were administered the Rapid Automatised Naming (RAN) task [53], Words Reading & Pseudoword Reading [54] and Text Reading [55] tasks to evaluate “overt” (i.e., aloud) reading abilities. Participants’ cognitive abilities were also assessed via the simple repetition (forward), backward repetition and reordering subtasks of the Digit Span from the WAIS-IV test battery [56] and Phonological Short Term Memory test [57, 58]. Specifically for Text Reading and Phonological Short Term Memory tests, alternative versions were used across sessions to minimise possible test–retest effects. These measures were included to ensure a comprehensive assessment of the impact of the interventions on both visual and auditory domains, also allowing us to investigate potential cross-domain effects of visuoattentional training and neuromodulation on the core functions impaired by DD. In order to control for possible differences in IQ between groups, which may have influenced training outcomes, participants' non-verbal IQ was assessed at baseline, during the first assessment session, by using Raven’s Advanced Progressive Matrices (APM) [59] (See Table 1).

#### EEG assessment of coherent motion perception and lexical access

**Fig. 2:**
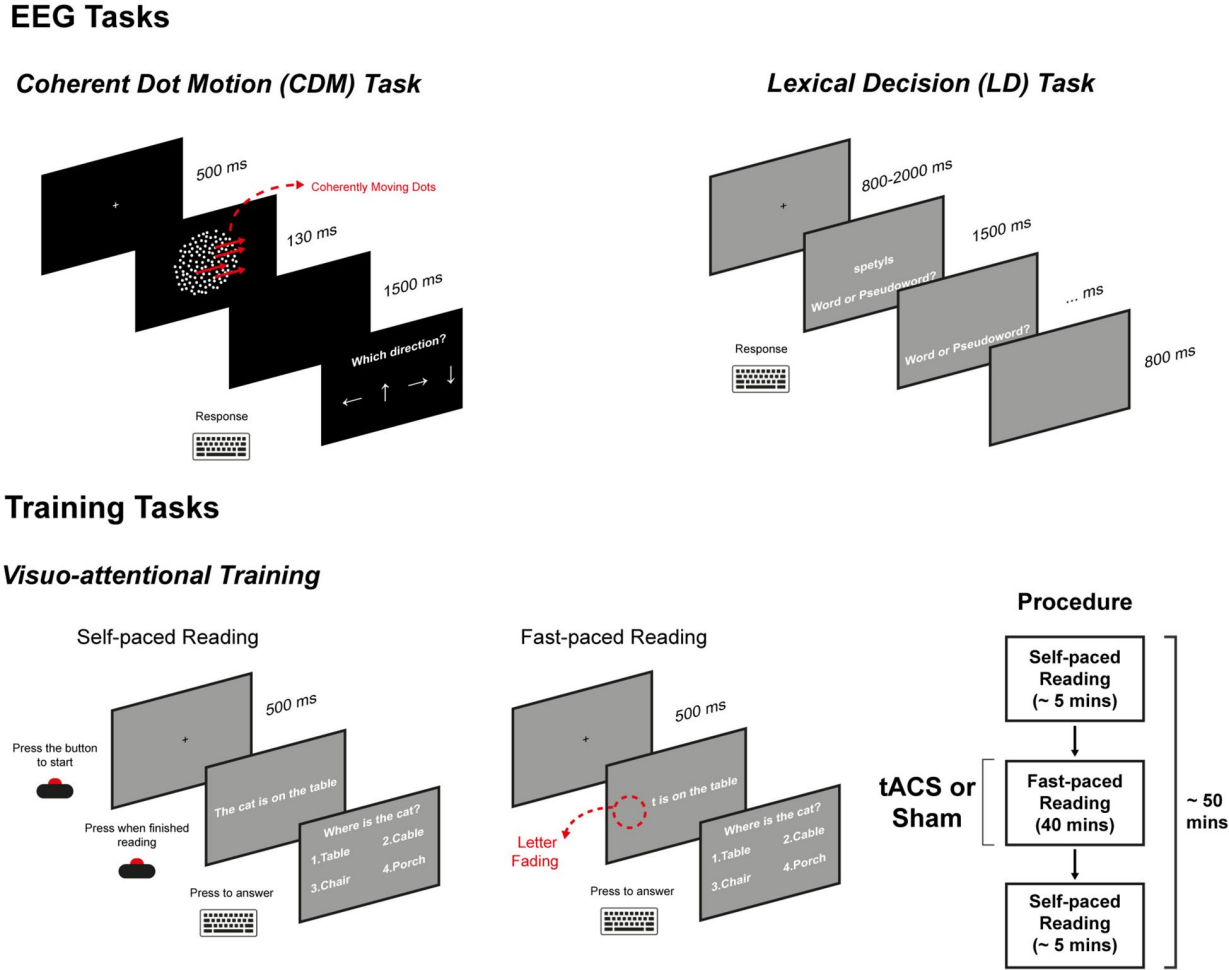
EEG Assessment tasks and training tasks. The picture depicts the EEG assessment tasks (Coherent Dot Motion and Lexical Decision) performed at T0 and T1 as well as the subtasks of the visuoattentional training

Coherent motion perception and lexical access were assessed via the Coherent Dot Motion (CDM, [60]) and the Lexical Decision (LD) [61] tasks. During both tasks, the EEG was recorded with a BrainProducts actiChamp + system at 1000 Hz sampling frequency from 64 Ag/Agcl shielded electrodes referenced to FCz placed in the standard 10–10 locations on an elastic cap. Impedance was kept < 15 kΩ. Both computerised tasks were run on a 240 Hz 54.5 × 30 cm screen (1920 × 1080 resolution) positioned at ~ 90 cm from the participant in a dimly lit room. The CDM task was run on MATLAB [62] via the Psychtoolbox toolbox [63] while the LD was run on Psychopy [64]. In each trial of the CDM task participants were presented with a fixation cross on a grey background in the centre of the screen for 500 ms. Then, white dots (*N* = 400, density = 9 dots/deg^2^) subtending a visual angle of 0.05 deg appeared on the screen enclosed in a 7 deg radius circle. The dots randomly moved in all directions at 13 deg/s speed for 128 ms. Each dot had a lifetime of 48 ms after which it reappeared in a different position. A proportion of the dots (Low Coherence = 1%, Mid Coherence = 10%, High Coherence = 20%) moved coherently in the same direction (Left, Right, Upward, Downward). After this, a blank screen was presented for 1500 ms. In a subsequent response screen, participants were asked to report in which direction they saw some of the dots moving coherently using the arrow keys on the keyboard (Fig. 2). The CDM task included 312 trials, equiprobably distributed across the design cells, and lasted ~ 20 min.

In each trial of the LD task, participants were presented with a fixation cross on a grey background with a jittered duration (Range = 800—2000 ms) at the centre of the screen. Then a word or a pseudoword was presented at the centre of the screen and the participants were asked to report whether they saw a word that was or was not in the Italian vocabulary using a button box. Response mapping was counterbalanced across participants. After 1500 ms the stimulus disappeared but participants could still provide a response. After the response a blank screen was presented for 800 ms. Two lists of 150 words and 150 pseudowords each were created by subsampling a bigger pool of stimuli drawn from previous studies using the same task [65]. In each list, pseudoword stimuli were created by minimally altering word stimuli. The same stimuli was used for all participants, and the presentation order was counterbalanced across participants. The task lasted ~ 20 min.

#### EEG recording and tACS administration during training

Eyes-closed Resting State EEG (RS-EEG) was recorded for 4 min from all participants with the eyes closed to reduce sensory inputs and artefacts (e.g., blinks, saccades), ensure a higher signal-to-noise ratio [66, 67] and foster comparability of results with previous studies [30, 31]). This was done with a StarStim system (Neuroelectrics, Inc.) using a neoprene cap with 32 electrodes before and after each training session. We used 23 Ag/AgCl EEG-only electrodes and 9 Ag/AgCl hybrid tACS/EEG electrodes (1 cm radius). Stimulation electrodes were placed in P4 and P3, while return electrodes were placed in C4, P8, O2, Pz, C3, P7, O1. All the remaining electrodes were used only for EEG recording. This bi-focal high-definition montage [31] optimally stimulated the parietal cortices (Fig. 1). Peak-to-baseline intensity of the stimulating electrodes was set at 0.8 mA with 0° phase, while the intensity of return electrodes was set to 0.228 mA with 180° phase. The frequency was set at 18 Hz based on previous studies [30, 31, 36]. Stimulation intensity was chosen following the tACS safety guidelines [45]. The total dosage of the stimulation protocol was 2749.2 mC (microCoulomb) for 40 min of stimulation for the Vis-tACS group. In the Vis-sham protocol, beta-tACS stimulation was delivered only for 30 s at the beginning and at the end of the task with ramped-up/down intensity. After each training session, all participants completed a questionnaire to evaluate their sensations during the stimulation [68] (see Supplementary Materials).

#### Visuoattentional training

The visuoattentional training was administered using the same apparatus employed in the CDM/LD tasks. For each session, after the first RS-EEG recording, participants were seated at ~ 90 cm from the screen in a dimly lit room and positioned their heads on a chin rest. In addition, an EyeLink 1000 eye-tracker (SR-Research) was mounted at ~ 60 cm from the participants’ chinrest. First, a 9-point gaze calibration procedure was performed. After this, the visuoattentional training session started. In the ‘self-paced’ reading procedure (~ 5 min), for each trial (*N* = 15) participants were presented with a sentence at the centre of the screen and were requested to read it silently. Sentences were presented in “Consolas” black monospaced font (letter width = 0.45 deg) and appeared when participants pressed a button on the button box. To ensure that participants would start reading from the start of the string, a “#” character was presented for 500 ms in the immediate left of the first character of the sentence before the sentence would appear. Once they finished reading, participants pressed a button on a button box and a 4-choice comprehension question appeared to verify that participants read the sentence. The comprehension questions simply enquired about the content of the sentence and did not require any reasoning. After they responded using the button box, the subsequent trial started. In the ‘fast-paced’ reading procedure (40 min), participants performed the same task, but in this case sentences were progressively faded from left to right by a sliding occluder [69]. Fading speed was initially set at the reading speed recorded during the self-paced reading procedure. Then, after 10 trials, it was adjusted based on the performance of the comprehension questions. Fading speed increased if the accuracy calculated on the previous 10 trials was 100%, it was kept constant if accuracy was between 80 and 100%, and it was diminished if accuracy was minor to 80%. After this, participants completed another self-paced reading procedure (~ 5 min). Throughout each session, the 9-point gaze calibration procedure was repeated other 3 times: before and in the middle of the fast-paced procedure, and before the second self-paced procedure. During the fast-paced reading procedure, beta-tACS was delivered to the Vis-tACS group while the Vis-Sham group received a placebo stimulation. Sentence stimuli along with the comprehension questions and response alternatives were drawn from a database of 2082 entries specifically developed for the present study (https://osf.io/exc73/?view_only=fd1622298c1e441fbab111bbcf0c3e1a).

Both self-paced and fast-paced procedures probe “covert” (i.e. silent) reading abilities. While overt reading abilities – assessed via neuropsychological tests – are essential for learning grapheme-to-phoneme mapping [70, 71], covert reading is the most ecological and used form of reading in everyday life of young adults.

On the one hand, the self-paced reading procedure (during which no stimulation was delivered) administered before every fast-paced reading procedure, served as an ecological assessment of covert reading abilities, allowing us to monitor possible training- or tACS-induced plastic changes occurring from session to session, net of the influence deriving from within-session training/tACS effects. On the other hand, the self-paced reading procedure administered after the fast-paced reading procedure had the purpose of evaluating short-term effects induced within each session right after the training/tACS.

The fast-paced reading procedure served as a training task in which participants were constantly challenged as fading speed continuously changed based on performance, representing an ideal target for tACS as the outcomes of its application depend on the behavioural and neural state during the stimulation [72–74]. That is, like the majority of neuromodulation techniques, tACS shows its full potential when the brain is engaged in a task which depends on neural oscillations within a specific frequency band, targeted by tACS. Moreover, combining tACS to a training procedure favours its neuroplastic effect on the brain areas/networks involved in the training task [75]. Accordingly, we hypothesised that beta-tACS would lead to reading performance improvements: i) while being applied during the fast-paced reading procedure (i.e., “online” effects), considering previous studies showing behavioural improvements at tasks probing cognitive/perceptual processes connatural to reading [30, 31, 76],ii) after its application across sessions (i.e., “offline”) effects, considering its ability to induce neuroplastic changes in the brain supporting long-term improvements [75, 77, 78].

#### Data analysis

##### Neuropsychological tests

Data from neuropsychological tests were first z-transformed or weighted based on the population norms when available, otherwise raw scores were used. Data were analysed by means of linear mixed models with the package lmerTest [79] in R [80]. All the models included *Group* (Vis-tACS, Vis-Sham) as categorical predictor and *Testing Time* as continuous predictor indicating the exact number of days passed from the first testing session averaged across participants The fixed effects as well as all the possible interactions between these predictors were estimated. Random intercepts for participants were also included. *Testing Time* was included as a 2nd degree polynomial and for each model the polynomial predictor led to lower AIC with respect to models with a simple linear predictor. When needed, post-hoc tests were run with the R package emmeans [81] and *p*-values were corrected with False Discovery Rate adjustment (FDR) [82]. Post-hoc tests performed to test the effect of *Testing Time* and its interactions were executed on Estimated Marginal Means (EMMs) considering the mean number of days passed from the first session across participant for each *Testing Time* (T0: 1 ± 0, T1: 40.40 ± 9.80, T2: 69.00 ± 10.40, T3 = 221 ± 12.2).

##### Visuoattentional training

For the reading speed measured during the visuoattentional training, the letter reading time (ms) of the trials in which participants correctly answered the comprehension questions was calculated for the self-paced procedures by dividing the total reading time by the number of letters in the sentence. For the fast-paced procedure, letter fading time was calculated for all trials as the time needed to occlude one single letter in the same way. Letter reading time (log) was analysed by means of linear mixed models with the package lmerTest in R. The model on the self-paced trials included *Group* (Vis-tACS, Vis-Sham), *Recording time* (Pre, Post) as categorical predictors and *Training session* (1–12, scaled) and the *Initial reading time* (scaled) recorded during the first self-paced procedure at the start of the training as continuous predictors. The fixed effects as well as all the possible interactions between these predictors were estimated. Random intercepts for participant and item (i.e., sentence) were also included. The model for the fast-paced trials was run on the letter fading time (log) and included the same predictors as the model run on self-paced data except for Recording time, which was not applicable in this case. Fixed effects were evaluated by Chi-square Anova tests with the Anova function of the “car” R library. When needed, post-hoc tests were run with the R package emmeans. FDR correction was applied for multiple comparisons. When interaction effects included the continuous covariate *Initial reading time*, EMMs or Estimated Marginal Trends (EMTs) for other categorical or continuous predictors included in the interaction were calculated on the 1 st and 3rd quartiles of *Initial reading time* leading to the subcategorisation of data points into “slow” and “fast” readers.

Eye-tracking data were preprocessed with the package eyelinkReader [83] in R. First, interest areas were defined for each word of each stimulus sentence as a rectangular area containing the word. Secondly, the number of regressive saccades, defined as saccadic movements initiated in an N interest area and terminated in an N-1 interest area, was computed for each sentence in each trial. Then, the proportion of regressive saccades over total saccades was analysed by means of generalised linear mixed models using a binomial linking function (logit) but separately for the self-paced and fast-paced procedure, as done for letter reading time. The model on the self-paced procedure included *Group* (Vis-tACS, Vis-Sham), *Recording time* (Pre, Post) as categorical predictors and *Training session* (1–12, scaled) as continuous predictor. Fixed effects were evaluated by Chi-square Anova tests with the Anova function of the “car” R library. When needed, post-hoc tests were run with the R package emmeans. FDR correction was applied for multiple comparisons.

##### Resting-state EEG

RS-EEG data recorded before and after each training session were preprocessed in MATLAB [62] using the EEGLAB [84] and FieldTrip [85] toolboxes. Data were resampled to 250 Hz and then pass-band (0.05—80 Hz) and notch (50 Hz) filtered. Data were segmented into 1-s epochs and those with signal amplitude surpassing ± 100 µV were marked for rejection. Channels responsible for the marking of more than 50% of total epochs were marked for rejection. Raw data were then reuploaded in MATLAB and previously marked channels were interpolated. Data was then re-referenced to the average reference, refiltered using the same filters mentioned above and re-epoched. Independent Component Analysis (ICA) was run on data and independent components were automatically labelled using ICLabel [86]. Components identified as muscle or eye activity with > 90% accuracy were removed. Epochs surpassing [−100 100] µV threshold were removed (Mean rejected epochs = 5.3 ± 0.5%). Next, the power spectrum was computed between 0 and 40 Hz with the fast Fourier Transform with multitaper estimation. The “Fitting Oscillations and One-Over-f'' (FOOOF) [87] toolbox was used to separately model the periodic (oscillatory) and the aperiodic power spectra. This toolbox allows for the computation of purely oscillatory activity by correcting for the aperiodic activity which is responsible for the 1/f power decay [87]. Beta power was computed from the periodic spectrum by averaging power between 15–25 Hz for all channels for each individual participant. The Individual Beta Frequency (IBF) was computed as the frequency with the highest power within the beta band for every channel and for each individual participant. Beta power and IBF data were extracted from the parieto-occipital channels (P3, P4, O1, O2, PO7, PO8, PO3, PO4, POz, Pz, Oz, P7, P8). The channel selection was based on previous studies showing: i) modulations of parieto-occipital beta oscillations related to visuoattentional processes [30, 31, 35, 36, 40], ii) anomalies in parieto-occipital beta oscillations in adults with DD [43], iii) modulations of parieto-occipital beta oscillations following the same beta-tACS protocol employed in the present study [30, 31].

Data were analysed by means of linear mixed models using the package lmerTest in R. Each model included *Group* (Vis-tACS, Vis-Sham), *Recording time* (Pre, Post), Channel (P3, P4, O1, O2, PO7, PO8, PO3, PO4, POz, Pz, Oz, P7, P8) as categorical predictors and *Training session* (1–12, scaled) as continuous predictor. The fixed effects as well as all the possible interactions between these predictors were estimated. Random intercepts for each participant were also included. Fixed effects were evaluated by Chi-square Anova tests with the Anova function of the “car” R library. When needed, post-hoc tests were run with the R package emmeans. FDR correction was applied for multiple comparisons.

##### Behavioural and EEG data of coherent dot motion and lexical decision task

Behavioural data from the CDM and LD tasks were analysed by means of generalised linear mixed models using a binomial linking function (logit). The model on CDM data included *Group* (Vis-tACS, Vis-Sham), *Testing Session* (T0, T1) as fixed effects as well as their interaction. Random intercepts and session slopes for each participant were also included. The model on LD data included *Group* (Vis-tACS, Vis-Sham), *Testing Session* (T0, T1) and *Lexicality* (word, pseudo-word) as fixed factors as well as all the possible interactions between them. Random intercepts and session slopes for each participants were also included. For both models, fixed effects were evaluated by Chi-square Anova tests with the Anova function of the “car” R library. When needed, post-hoc tests were run with the R package emmeans. FDR correction was applied for multiple comparisons.

EEG data from CDM and LD tasks were preprocessed in MATLAB using the EEGLAB and ERPLAB [88] toolboxes. Data were resampled to 250 Hz and then pass-band (0.05—80 Hz) and notch (50 Hz) filtered. Data were segmented in epochs from −200 to 1200 ms relative to stimulus onset and baseline correction was applied in the −200 to 0 ms interval. Epochs with EEG surpassing ± 300 µV were marked for rejection. Channels responsible for the marking of more than 20% of total epochs were marked for rejection. Raw data were then reuploaded in MATLAB and previously marked channels were interpolated. Data was then re-referenced to the average reference and refiltered using the same filters mentioned above. Independent Component Analysis (ICA) was run on data and independent components were automatically labelled using ICLabel. Components identified as muscle or eye activity with > 90% accuracy were removed. Epochs surpassing ± 100 µV threshold were removed.

Previous literature showed that attenuated early ERPs in the low (but not in the mid and high) coherence condition during coherent motion discrimination correlate with the magnitude of literacy deficit in developmental dyslexia [89]. Thus, we selected low coherence (1%) trials to investigate evoked responses during the CDM task administered before and after the training.. ERPs were computed from 200 ms prior to stimulus onset to 800 ms after stimulus onset. First, ERPs were segmented into specific time windows for each component of interest (i.e., P1: 90–130 ms, N2: 130–200 ms, P2: 200–270 ms). Then, mean amplitude values (i.e., microvolts) were extracted for each participant and each session from P3 and P4 electrodes. Finally, a mixed-design analysis of variance with Group (Vis-tACS, Vis-Sham) as a between-group factor and Testing Session (T0, T1) and Hemisphere (left, right) as within-group factors was performed on for each component using the ‘ez’ [90] package in R. Paired-sample t tests (planned contrasts) were used to further investigate significant ANOVA’s interactions using the ‘stats’ package. False Discovery Rate (FDR) correction was applied to control for multiple comparisons.

For the lexical decision task, P300 and N400 ERP components were analysed to assess two successive processing stages of language processing. Mean amplitude of P300 was extracted from a central-parietal electrodes cluster, comprising Cz, CPz, C1, C2, CP1, CP2 channels in the 280–380 ms time window; mean amplitude of N400 was extracted at a frontal-central electrodes cluster, comprising Fz, FCz, F1, F2, FC1, FC2 channels in the 350–450 ms time window. The choice of the electrode clusters and time windows were made based on previous literature [91–93]. The mean amplitude values of each ERP component underwent mixed-design ANOVAs with *Group* (Vis-tACS, Vis-Sham) as between-group factor, *Testing session* (T0, T1) and *Lexicality* (word, pseudo-word) as within-group factors. ANOVAs were computed using ez package in R; paired-sample t tests (planned contrasts) were used to further investigate significant effects. FDR correction was used to control for multiple comparisons. Greenhouse–Geisser correction for sphericity was applied when needed.

##### tACS safety and tolerability

To evaluate safety and tolerability of multiple sessions of tACS, sensations referring to different perceptions of discomfort (i.e., itching, pain, burning, heat, pinching, iron taste, and fatigue) were collected at the end of each experimental session [68]. The proportion of reported sensation was mediated across sessions and compared between the 2 groups using Kruskal–Wallis chi-squared test (see Supplementary Materials). To assess the blinding quality of the sham/placebo groups, the proportion of participants who correctly identified their group assignment was compared between Vis-tACS and Vis-Sham groups using Kruskal–Wallis chi-squared test. This analysis was conducted on the perceived allocation responses collected at T3 (active, placebo, or "don't know"). In the Results section only the most salient effects are reported. The remaining effects emerging from omnibus tests are reported in Supplementary Materials.

## Results

First, we evaluated the impact of bilateral parietal beta tACS, combined with the visuoattentional reading acceleration training, on reading and reading-related cognitive functions. Next, we focused on the tACS-induced enhancement of reading skills and oculomotor control by comparing speed, accuracy and regressive saccades obtained during computerised self-paced reading between the Vis-tACS and the Vis-Sham groups across all training sessions (1–12).

Furthermore, we explored the tACS-induced plastic changes at resting state EEG (RS-EEG) looking at modifications in oscillatory activity across all groups throughout the 12 training sessions. Finally, we investigated possible tACS-induced neurophysiological enhancements on lexical processing (lexical decision, LD) and coherent dot motion (CDM) tasks by comparing event-related potentials (ERPs) across the two groups and testing sessions (T0, T1).

### Beta-tACS selectively boosts working memory while showing equal improvements in standardised reading tests

Reading speed measured with standardised tests improved for all groups, showing a *Testing session* effect, for text (χ^2^(2) = 24.48, *p* < 0.001; T0 vs T1 = −0.87, SE = 0.18, *p*_FDR_ < 0.001; T0 vs T2 = −1.33, SE = 0.27, p_FDR_ < 0.001; T0 vs T3 = −1.058, SE = 0.28, p_FDR_ < 0.001), words (χ^2^(2) = 64.41, *p* < 0.001; T0 vs T1 = −0.46, SE = 0.07 p_FDR_ < 0.001; T0 vs T2 = −0.72, SE = 0.10, p_FDR_ < 0.001; T0 vs T3 = −0.76, SE = 0.10, p_FDR_ < 0.001) and pseudowords (χ^2^ (2) = 70.68, *p* < 0.001; T0 vs T1 = −0.347, SE = 0.04ì6, p_FDR_ < 0.001; T0 vs T2 =—0.59, SE = 0.09, p_FDR_ < 0.001; T0 vs T3 = −0.72, SE = 0.09, p_FDR_ < 0.001) reading. Post-hoc performed on all reading tests showed that all participants improved their speed at all reading tests and their improvement lasted at least until 6 months after the training.

Phonological processing skills measured with Rapid Automatized Naming (RAN) test also improved after the training, showing a *Testing session* effect (χ^2^(2) = 13.27, *p* = 0.001; T0 vs T1 = 0.58, SE = 0.17, p_FDR_ < 0.001; T0 vs T2 = 0.89, SE = 0.26, p_FDR_ < 0.001; T0 vs T3 = 0.82, SE = 0.26, p_FDR_ = 0.001) Finally, regarding the digit span test, a *Testing session* effect was found for the standardised score calculated from all subtests (χ^2^(2) = 13.13, *p* = 0.001; T0 vs T1 = −1.07, SE = 0.30, p_FDR_ < 0.001; T0 vs T2 = −1.62, SE = 0.45, pFDR < 0.001; T0 vs T3 = −1.01, SE = 0.406, p_FDR_ = 0.017) and for the simple repetition subtest (χ^2^(1) = 13.30, *p* = 0.001; T0 vs T1 = −0.85, SE = 0.23, p_FDR_ < 0.001; T0 vs T2 = −1.29, SE = 0.36, p_FDR_ < 0.001; T0 vs T3 = −0.79, SE = 0.36, p_FDR_ = 0.015). A *Group x Testing Session* Interaction in the reordering subtest (χ^2^(2) = 6.19, *p* = 0.045) showed an improvement in working memory lasting up until 6 months, only for the Vis-tACS (T0 vs T1 = 1.22, SE = 0.34, p_FDR_ = 0.001; T0 vs T2 = 1.85, SE = 0.52, p_FDR_ = 0.001; T0 vs T3 = 1.06, SE = 0.51, p_FDR_ = 0.043). No significant training effects emerged for the backward repetition subtest (all ps > 0.56), for the phonological short-term memory test (all ps > 0.29), and for text (all ps > 0.49), words (all ps > 0.18), pseudowords (all ps > 0.27) reading accuracy.

**Fig. 3:**
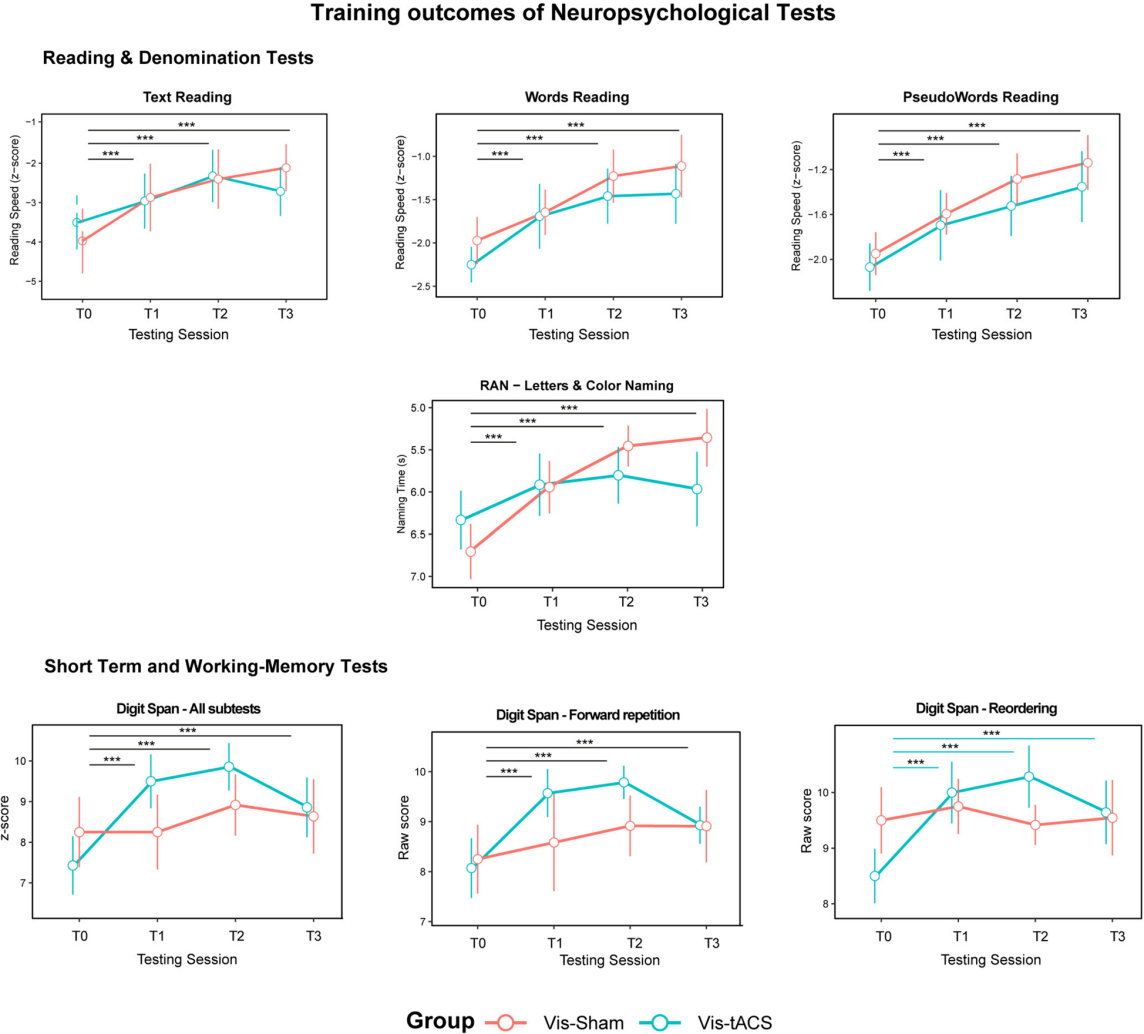
Training outcomes of neuropsychological tests. The picture summarises the relevant outcomes obtained at neuropsychological tests for each group (Vis-Sham in red, Vis-tACS in blue) and testing session (T0, T1, T2, T3). Dots represent the average of the dependent variable indicated on the y-axis along with SEM bars separately for each group and testing session (x-axis). For Text, Words and Pseudowords reading tests (1st row), as well as for the Digit Span—All subtests (3rd row), the y-axis shows the standardised score, while for the RAN test (2nd row) and the Forward and Reordering subtests of the Digit span (3rd row) shows raw scores. Statistical significance is indicated by horizontal bars and asterisks (*p* <.05 *, *p* <.01 **, *p* <.001***); black bars refer to post-hoc tests operated irrespectively of the group to explore the Testing Session effect while coloured bars refer to post-hoc test performed in a specific group to explore Group x Testing Session interactions

These results show that visuoattentional training irrespectively of beta-tACS lead to improvements in ‘overt’ reading skills, as assessed by standard neuropsychological tests, lasting up to 6 months after the training. Importantly, only beta-tACS specifically induced a long-lasting improvement in working memory for the same timeframe (Fig. 3).

### Beta-tACS selectively improves ‘covert’ reading speed and oculomotor efficiency

For self-paced reading speed measured at each training session, a *Group x Training session* interaction (χ^2^ (1) = 7.93, *p* = 0.005) showed that the letter reading time diminished faster for the Vis-tACS group (Slope = −0.211, SE = 0.010) across the 12 sessions with respect to the Vis-Sham group (Slope = −0.164, SE = 0.10; Vis-tACS vs Vis-Sham = 0.047, SE = 0.014, *p*_FDR_ < 0.001).

**Fig. 4:**
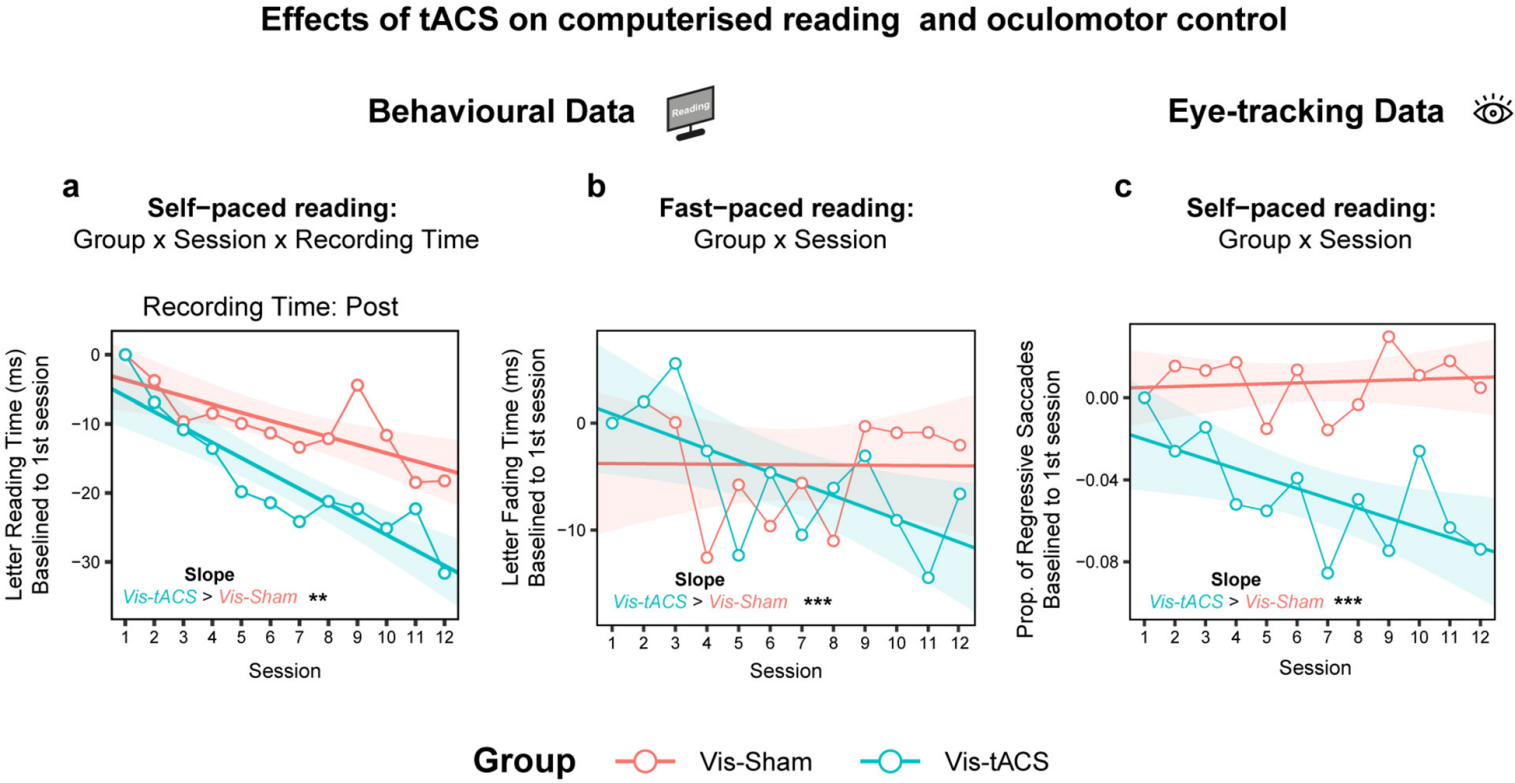
Effects of tACS on reading and oculomotor control. **a** Letter reading time (ms) baselined to the 1 st session (y-axis) recorded during the self-paced reading procedure after each one of the 12 sessions (x-axis) of the visuoattentional training for the Vis-Sham (red) and Vis-tACS (blue) groups. Line-connected dots represent letter reading time averaged across individual participants separately for each group along with the linear fit (shaded areas indicate SEM). **b** Letter fading time (ms) baselined to the 1 st session (y-axis) recorded during the fast-paced reading procedure of the 12 sessions (x-axis) of the visuoattentional training for the Vis-Sham (red) and Vis-tACS (blue) groups. Line-connected dots represent letter reading time averaged across individual participants separately for each group along with the linear fit (shaded areas indicate SEM). **c** Proportion of regressive saccades over total saccades baselined to the 1 st session (y-axis) recorded during the self-paced reading procedure before and after (averaged) each one of the 12 sessions (x-axis) of the visuoattentional training for the Vis-tACS (blue) and Vis-Sham (red) groups. Line-connected dots represent the proportion of regressive saccades averaged across individual participants separately for each group along with the linear fit (shaded areas indicate SEM). Statistical significance is indicated by asterisks (*p* <.05 *, *p* <.01 **, *p* <.001***) near text labels in each subplot specifying the direction of tests’ results

The *Group x Training session x Recording time* interaction (χ^2^(1) = 5.19, *p* = 0.023) indicated that letter reading time recorded during self-paced reading before each training session diminished with similar speed both in the Vis-tACS (Slope = −0.215, SE = 0.014) and the Vis-Sham group (Slope = −0.199, SE = 0.015; Vis-tACS vs Vis-Sham = 0.015, SE = 0.019, p_FDR_ = 0.56), while letter reading time recorded after each training session diminished faster for the Vis-tACS group (Slope = −0.208, SE = 0.014) with respect to Vis-Sham group (Slope = −0.129, SE = 0.015; Vis-tACS vs Vis-Sham = −0.079, p_FDR_ = 0.001) (Fig. 4a). These effects highlight an ‘offline’ advantage of the beta-tACS that extends beyond the stimulation period itself, leading to more pronounced benefits in reading speed across sessions.

The *Group x Training session x Initial reading tim*e interaction (χ^2^(1) = 262.61, *p* < 0.001) further indicated that letter reading time diminished faster in slower Vis-tACS readers (Slope = −0.308, SE = 0.011) with respect to slower Vis-Sham readers (Slope = −0.181, SE = 0.011; Vis-tACS vs Vis-Sham = 0.127, SE = 0.016, p_FDR_ < 0.001).

These results show that beta-tACS boosted the self-paced reading speed enhancement induced by the training, as outlined by steeper improvement trajectories. Secondly, the tACS-induced improvement was more pronounced when looking at self-paced reading speed after each training session, and also more pronounced for slower readers.

When analysing fading speed recorded during fast-paced reading, a *Group x Training session* interaction (χ^2^(1) = 11.49, *p* < 0.001) showed that the Vis-tACS group (Slope = −0.090, SE = 0.005) achieved faster acceleration across the 12 sessions with respect to the Vis-Sham group (Slope = −0.065, SE = 0.006; Vis-tACS vs Vis-Sham = 0.025, SE = 0.008, p_FDR_ = 0.001; Fig. 4b). The interaction *Group x Training session x Initial reading time* (χ^2^(1) = 506.201, *p* < 0.001) showed that, similarly to what was observed for self-paced reading, letter fading time diminished faster across training sessions for Slow Vis-tACS readers (Slope = −0.201, SE = 0.006) with respect to Slow Vis-Sham readers (Slope = −0.069, SE = 0.007; Vis-tACS vs Vis-Sham = 0.132, SE = 0.010, p_FDR_ < 0.001).

Crucially, regarding reading-related oculomotor efficiency, the *Group x Training session* interaction (χ^2^(1) = 75.64, *p* < 0.001) showed that the proportion of regressive saccades diminished only in the Vis-tACS group (Slope = −0.46, SE = 0.036) but not in the Sham group (Slope = 0.02, SE = 0.04; Vis-tACS vs Vis-Sham = 0.48, SE = 0.05, p_FDR_ < 0.001) (Fig. 4c). These effects indicate that beta-tACS induced a significant reduction of regressive saccades with respect to the visuoattentional training alone. Of crucial relevance, the tACS-induced reduction of regressive saccades corroborates the improvement of self-paced reading speed and suggests that optimal oculomotor control has a primary role in supporting efficient reading and training-induced reading improvements.

Overall, these results highlight both ‘online’ (i.e., during fast-paced reading) and ‘offline’ (i.e., during self-paced reading) benefits of beta-tACS, which allow individuals with DD to read accurately even with high fading speeds.

### Parietal beta-tACS favours plastic changes in the brain of adults with developmental dyslexia

**Fig. 5:**
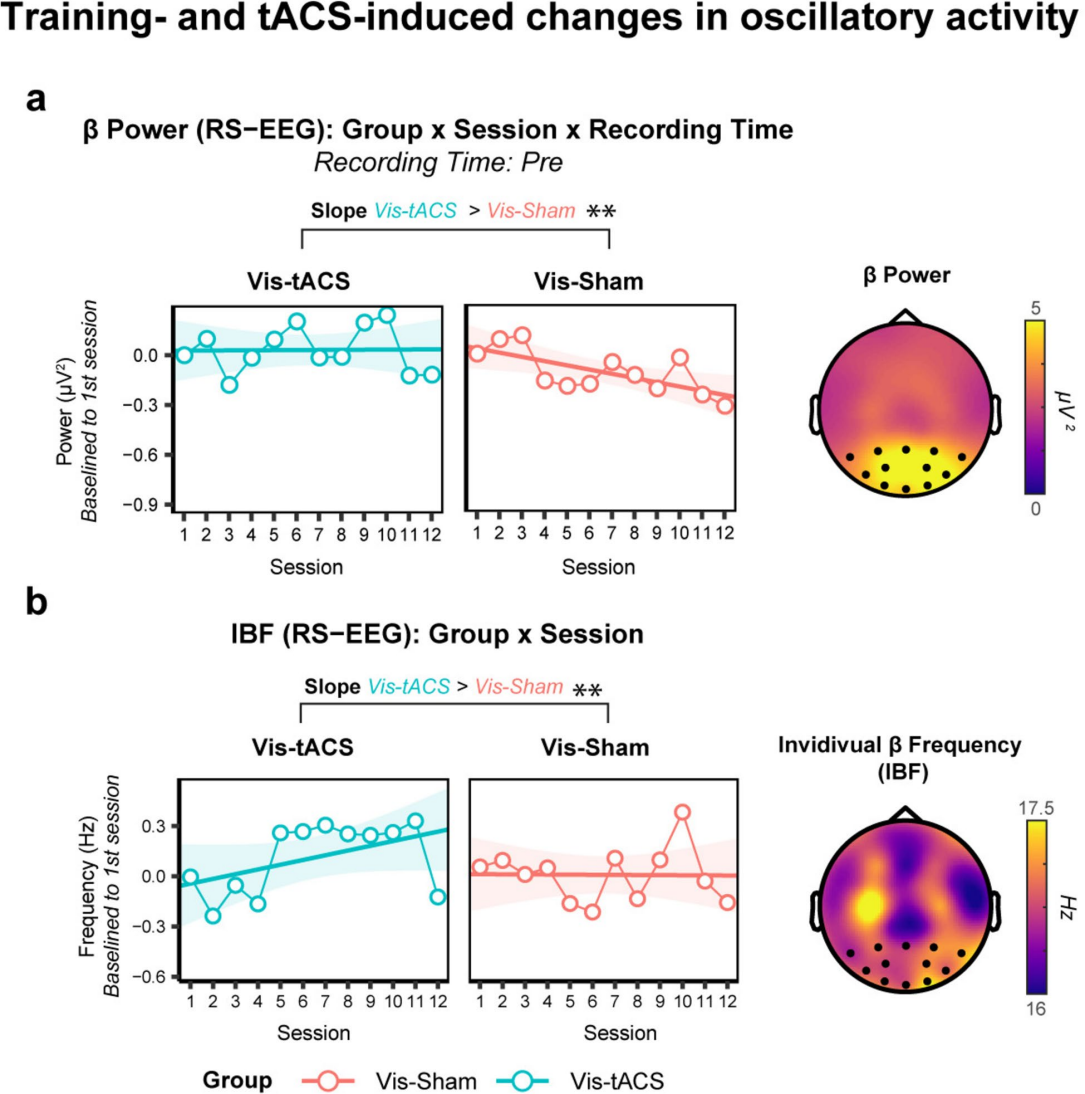
Training- and tACS-induced changes in oscillatory activity. **a** Line-connected dots represent beta-power (μV^2^) baselined to the 1st session (y-axis) and averaged across individual participants, separately for each group (Vis-tACS in blue, Vis-Sham in red). Lines represent the linear fit (shaded areas indicate SEM) of the self-paced reading procedure recorded before the 12 sessions (x-axis) of the training. Topographical maps on the right depict the distribution of beta-power on the scalp of the first resting-state EEG (RS-EEG) recorded prior to the start of the training averaged across all participants and corrected for aperiodic activity via FOOOF algorithm (see Methods section). Black dots indicate the parieto-occipital channels used in statistical analyses. **b** Line-connected dots represent IBF (Hz) baselined to the 1st session (y-axis) and averaged across individual participants, separately for each group (Vis-tACS in blue, Vis-Sham in red). Lines represent the linear fit (shaded areas indicate SEM) of the self-paced reading procedure recorded before the 12 sessions (x-axis) of the training. Topographical maps on the right depict the distribution of IBF on the scalp of the first resting-state EEG (RS-EEG) recorded prior to the start of the training averaged across all participants and corrected for aperiodic activity via FOOOF algorithm (see Methods section). Black dots indicate the parieto-occipital channels used in statistical analyses. Statistical significance is indicated by black horizontal bars and asterisks above the subplots (*p* <.05 *, *p* <.01 **, *p* <.001***). The direction of tests’ results is indicated by text labels above black bars

When looking at beta power from RS-EEG, a *Group x Training session* interaction (χ^2^(2) = 8.47 *p* = 0.004) showed that beta power diminished across training sessions for the Vis-Sham group (Slope = −0.151, SE = 0.027, p_FDR_ < 0.001), while it remained relatively stable for the Vis-tACS group (Slope = −0.040, SE = 0.026, p_FDR_ = 0.130; Fig. 5a). The slope of the Vis-Sham group was significantly steeper with respect to the Vis-tACS (Vis-tACS vs Vis-Sham = 0.111, SE = 0.038, p_FDR_ = 0.014).

For Individual Beta Frequency (IBF) computed from RS-EEG, the *Group x Training session* interaction (χ^2^ (2) 10.34, *p* = 0.001) showed that IBF constantly increased across sessions for the Vis-tACS group (Slope = 0.303, SE = 0.065, p_FDR_ < 0.001), while it remained stable for the Vis-Sham (Slope = −0.005, SE = 0.070, p_FDR_ = 0.941; Fig. 5b). The increase in the Vis-tACS group was significantly different from the flat trend found in the Vis-Sham (Vis-tACS vs Vis-Sham = 0.309, SE = 0.096, p_FDR_ = 0.001). The analysis of beta-band oscillatory activity suggests that beta-tACS can induce specific plastic changes in the brain of individuals with DD as shown by the increment of parieto-occipital IBF, while keeping beta power levels stable in time.

### Parietal beta-tACS improves visual motion perception and induces more efficient lexical access

**Fig. 6:**
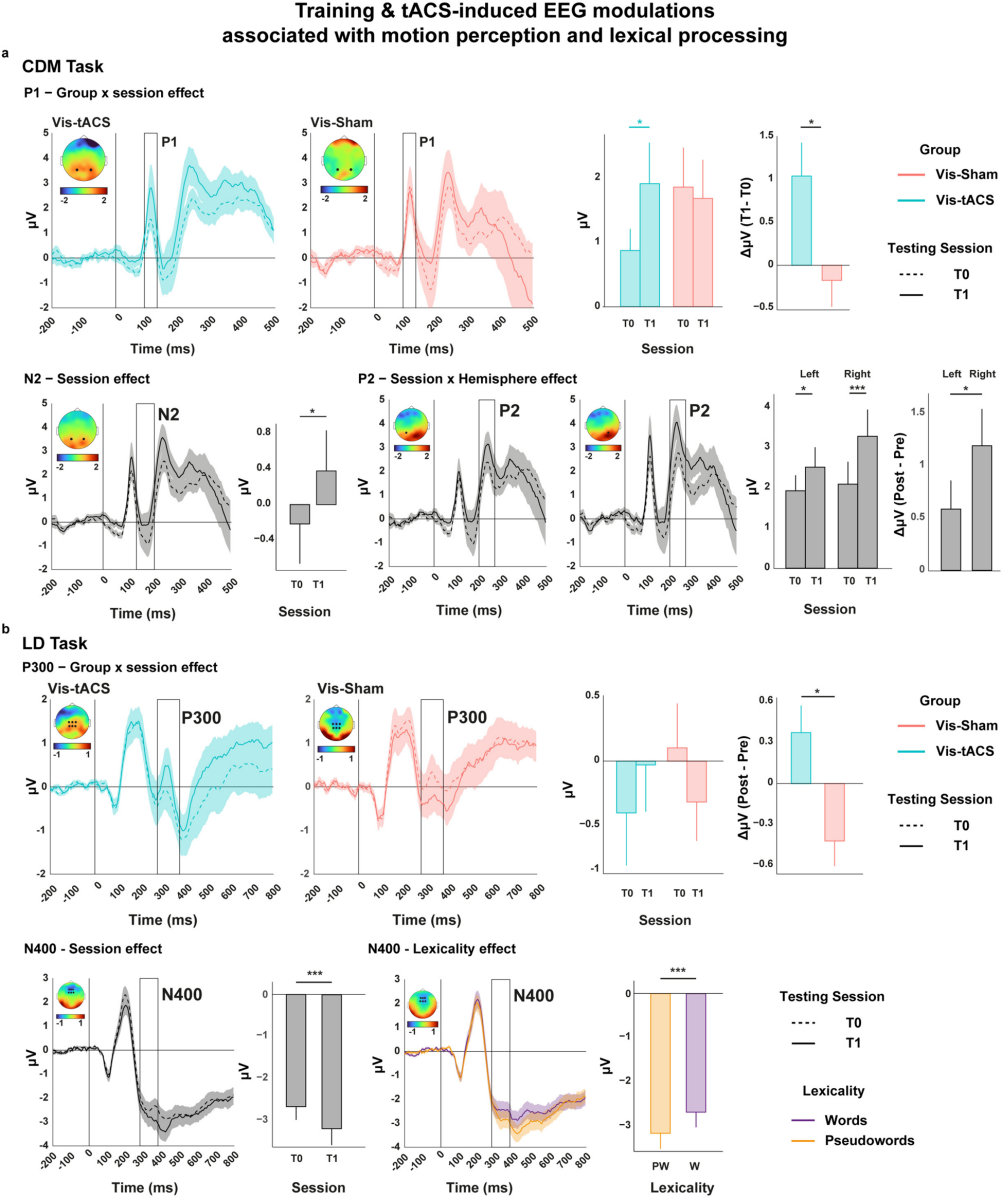
Training & tACS-induced EEG modulations associated with motion perception and lexical processing. **a** Lines represent ERPs waveforms with SEM (shaded areas) following the presentation of low-coherence (1% of moving dots) trials of the CDM task computed in the left (channel P3) and right (channel P4) hemisphere at T0 (dotted line) and T1 (solid line) testing session averaged across participants within each group groups (Vis-tACS in blue, Vis-Sham in red) or across all groups (black). Each subplot depicts a specific effect found for a specific ERP component. Black squares indicate the time window in which the mean amplitude of components P1, N2 and P2 was extracted for statistical analyses. Topographies represent the distribution on the scalp of the effect corresponding to the differences between the averaged ERP amplitudes of the corresponding waveforms (e.g., session T1—session T0). Black dots on the topography indicate the channels used for the analyses (P3, P4). Each subplot also contains barplots indicating the Mean and SEM of the design cells interested by each specific effect. Statistical significance is indicated by asterisks (*p* <.05 *, *p* <.01 **, *p* <.001***). (**b**) Lines represent ERPs waveforms with SEM (shaded areas) following the presentation of stimuli of the LD task at T0 (dotted line) and T1 (solid line) testing session averaged across participants within each group (Vis-tACS in blue, Vis-Sham in red), across all groups (black) or within trials in which words or pseudowords were presented (words in violet, pseudowords in orange). Topographies represent the distribution on the scalp of the effect corresponding to the differences between the averaged ERP amplitudes of the corresponding waveforms (e.g., session T1—session T0). Black squares indicate the time window in which the mean amplitude of components P300 and N400 was extracted for statistical analyses. Black dots on the topography indicate the channels used for the analyses (Cz, CPz, C1, C2, CP1, CP2 for the P300 and Fz, FCz, F1, F2, FC1, FC2 for N400). Each subplot also contains barplots indicating the Mean and SEM of the design cells interested by each specific effect. Statistical significance is indicated by asterisks (*p* <.05 *, *p* <.01 **, *p* <.001***)

Accuracy data from the CDM task revealed no effects (all ps >. 65). The ANOVA on the P1 (90–130 ms) mean amplitude measured during the CDM task showed a significant *Group x Testing session* interaction (F(1,25) = 5.52, *p* = 0.026, η_p_^2^ = 0.18). Post-hoc comparisons revealed that P1 grew significantly larger after the training (T1) in the Vis-tACS group (t(13) = 2.66, pFDR = 0.033, ΔM = 1.04 µV, SE = 0.38) while in the Vis-Sham group it did not (t(12) = −0.40, pFDR = 0.58, ΔM = −0.11 µV, SE = 0.28); the difference between T1 and T0 was also larger in the Vis-tACS group with respect to the Vis-Sham group (t(23.48) = 2.44, pFDR = 0.033; see Fig. 6a). Considering the N2 (130–200 ms) mean amplitude of the CDM task, only a significant main effect of *Testing session* emerged (F(1,25) = 6.07, *p* = 0.020, η_p_^2^ = 0.19) showing that the N2 component grew smaller after the training ΔM = 0.70 µV, SE = 0.27, See Fig. 6a).

The ANOVA on the P2 (200–270 ms) mean amplitude during the CDM task showed a significant *Testing session x Hemisphere* interaction (F(1,25) = 6.90, *p* = 0.014, η_p_^2^ = 0.21). Post-hoc comparisons showed that P2 significantly grew larger in both the left (t(26) = 1.79, pFDR = 0.04, ΔM = 0.58 µV, SE = 0.32) and the right (t(26) = 4.24, pFDR < 0.001, ΔM = 1.54 µV, SE = 0.36) hemispheres but way more on the right (t(26) = −2.66, pFDR = 0.019, ΔM = −0.96 µV, SE = 0.36, See Fig. 6a).

These results show a significant tACS-induced modulation of the P1 component and a training-induced modulation of the N2 and P2 components measured during coherent motion perception in both groups. It is noteworthy that the modulation of the P2 component was stronger in the right hemisphere.

Behavioural data from the LD task revealed a main effect of Lexicality χ^2^(1) = 63.38, *p* < 0.001, showing that participants were more accurate into recognising words (EMM = 0.98, SE = 0.002) with respect to pseudowords (EMM = 0.95, SE = 0.005). A Group x Category effect also emerged χ^2^(1) = 17.91, *p* < 0.001. Post-hoc tests revealed that the Vis-Sham group performed better on pseudowords (EMM = 0.96, SE = 0.005), with respect to the Vis-tACS group (EMM = 0.94, SE = 0.009; Vis-Sham vs Vis-tACS = 1.82, SE = 0.39, *p* = 0.009).

In the lexical decision task we analysed P300 and N400 to access, respectively, working memory and lexical processing efficiency. The ANOVA performed on the P300 mean amplitude showed a significant *Group x Testing Session* interaction (F (2,34) = 4.20, *p* = 0.023, ηp^2^ = 0.20). To further investigate this interaction, post-hoc comparisons on the difference in amplitude between T0 and T1 were computed, and then contrasted between groups. P300 was differently modulated in the Vis-tACS group, where it increased from T0 to T1 (ΔM = 0.373, eSD = 0.74), as compared to the Vis-Sham group, where it decreased from T0 to T1 (ΔM = −0.421, SD = 0.66) (*t*(24.9) = 2.94; pFDR = 0.02; Fig. 6b).

The ANOVA performed on the N400 mean amplitude showed significant effects of *Lexicality* (F(1,34) = 35.20, *p* < 0.001, η_p_^2^ = 0.51) and *Testing session* (F(2,34) = 8.46, *p* < 0.001, η_p_^2^ = 0.21). N400 was larger for pseudowords (M = −2.96 µV, SE = 0.22) as compared to words (M = −2.46 µV, SE = 0.22), and larger at T1 (M = −2.93 µV, SE = 0.24) as compared to T0 (M = −2.49 µV, SE = 0.20; Fig. 6b).

The P300 amplitude increment between T0 and T1, found only in the Vis-tACS group, reflects a tACS-induced enhancement of working memory capacity during lexical access [91, 92]. The N400 also increased in amplitude from T0 to T1, indicating a more effective retrieval of lexical information in all trained groups.

### Safety and tolerability of prolonged beta-tACS administration and quality of blinding

Kruskal–Wallis chi-squared test revealed that the reported sensations after beta-tACS or sham stimulation did not differ between groups (See Supplementary materials). The analysis on perceived allocation showed that the number of participants who correctly identified their group assignment (active vs placebo/sham) at T3 was not significantly different (χ^2^(1) = 1.30, *p* = 0.25). This result indicates that the blinding procedure was effective.

## Discussion

In the present study we proposed for the first time a multi-focal neuromodulation protocol specifically designed to improve the dorsal visual stream functionality in individuals with DD. We demonstrated its efficacy in ameliorating visual processes underlying reading, oculomotor efficiency and other DD-related core deficits in the long term, particularly working memory and visual motion perception. These benefits were accompanied by specific plastic changes in the neuromodulated brain areas. By encompassing the highest number of tACS sessions administered in a population affected by neurodevelopmental disorders to date, this study further remarks on the safety and tolerability of this technique when used in multi-sessions training protocols [45, 68, 94, 95]. Overall, these findings set a new promising and safe pathway for the treatment of visual deficits in DD and in other clinical conditions, showing that beta-tACS targeting parietal areas is an effective strategy for the (re)habilitation of different DD core deficits. Furthermore, our results represent the first evidence of a causal link between beta-band oscillations, with a main hub in superior parietal areas, and reading impairments in DD as proposed in previous studies [30, 31, 40, 43]. These findings not only provide key mechanistic insights of the neurobiological roots of visual impairments in DD, but also add compelling evidence on whether visual deficits are directly responsible for reading impairments.

Of primary clinical relevance, the concurrent improvements in reading speed and oculomotor efficiency induced by beta-tACS delivered on parietal sites testify the successful improvement in visuoattentional, visuospatial, and oculomotor processing that heavily impact reading in DD. The posterior parietal cortex has robust functional connectivity to frontal areas within the beta (15–25 Hz) band [96] and has an important role in saccades planning as it is essential to orient and maintain visuospatial attention [97–99], as well as to provide spatial coordinates to the Frontal Eye Fields (FEF) that are responsible for movement execution [100]. This chain of processes must be deployed with high speed and precision during reading while foveating from one word to the next [18]. The improvement in oculomotor efficiency observed here likely results from ameliorated visuospatial attention processes, typically impaired in DD [13, 24]. This in turn allowed for more efficient saccade programming [101–103], resulting in a reduction of regressive saccades [28, 29] and an optimization of information intake, ultimately leading to more fluent reading [18, 23].

The positive impact of beta-tACS on visuospatial processes was further corroborated by the neurophysiological improvements in visual motion processing, as indexed by the specific modifications of P1 neural component during visual motion perception [104] that were possibly not captured by behavioural data. P1 is typically associated with dorsal stream functionality [105] and its amplitude is enhanced by visuospatial attention during visual motion perception [106, 107]. Individuals with DD struggle with visual motion processing, in line with the anomalies along the dorsal stream [17, 108], showing attenuations in P1’s amplitude [89, 109]. Thus, the enhancement of P1 during motion perception following the visuoattentional training with parietal beta-tACS suggest improvements of visuospatial and motion perception abilities. The visuoattentional training also showed an amplitude decrement in the N2 and an increment in the P2 evoked by motion perception, irrespectively of beta-tACS. The N2 is associated with global motion integration and scales with attentional engagement [110, 111], while the P2 reflects higher-level evaluation of motion features [104] and top-down control in visual perception [112]. In this case, the visuoattentional training possibly lowered the amount of attentional resources required for motion processing while also favouring deeper and higher-level analysis of motion stimuli.

The present study further corroborates previous theoretical perspectives framing parietal beta oscillations as the key rhythm governing not only the dorsal-to-ventral visual streams communication which promotes visuospatial analysis [30, 31, 34–39], for a review see [40], but also the fronto-parietal dorsal communication which guides oculomotor control [32, 33, 96, 113]. Importantly, the session-by-session modulations of beta oscillations demonstrate that repeated beta-tACS application induces specific plastic changes in the brain, confirming the predictions made by previous single-session studies [31, 114–116]. In particular, parietal beta-tACS led to a session-by-session increase of IBF over parieto-occipital sensors. Beta oscillations are thought to mediate functional inhibition in the motor and perceptual domains [117]. Specifically, the central frequency of beta oscillations is a relevant mediator of the excitability of the sensorimotor system [118]. Secondly, the individual beta frequency, a stable trait-like measure of endogenous beta activity recorded at rest [119], is positively correlated with GABA concentration, the main inhibitory neurotransmitter in the central nervous system [120, 121]. The tACS-induced plastic changes of IBF together with visuospatial/visuoattentional and oculomotor improvements align well with the domain-specific and domain-general functional specifications of beta frequency reported in previous studies. Additionally, this result further acquires relevance considering the Neural Noise Hypothesis [122], which suggest that individuals with DD are characterised by neural over-excitation,increases in IBF could possibly indicate an increase in GABA-mediated inhibitory activity, balancing out over-excitation. The training itself also led to session-by-session neuroplastic changes as indicated by a decrease in beta power observed in the Vis-Sham group. Long-term reductions in beta power were observed following word learning [123] and intensive meditation [124], while short term increases were found following visuomotor training [125, 126], highlighting the importance of beta activity for learning and memory consolidation. The absence of the same pattern in the Vis-tACS group might simply emerge from beta-tACS compensating a power decrease with an increase fostered by neuronal entrainment [74]. However, it is important to recognise that, as shown by previous studies, the direction of tACS-induced modulations in spectral power recorded before and after stimulation are not always consistent [30, 31].

Apart from visuoattentional and phonological disruptions, working memory impairments in DD persist throughout adulthood, strongly impacting reading comprehension [127–129]. Therefore, tACS-induced enhancements of working memory lasting up to 6 months represent a decisive response to this substantial impairment in DD, [130–132]. This fundamental clinical outcome is in line with the well-known involvement of the parietal cortex in mnemonic functions [133–135], but is also crucial since it provides causal evidence of the association between beta band activity and working memory, suggested by recent experimental evidence [136, 137]. Poor working memory considerably affect phonological [132, 138] lexical [139] and visuoattentional skills [140] in DD. Hence, we investigated the P300 component, recorded during the lexical decision task, as a well-established neural index of working memory abilities [141–144]. Specifically, the P300 amplitude reflects the amount of the attentional resources deployed [145], WM updating efficiency [146, 147] and capacity [148]. However, when higher levels of automatisation are involved following training, the P300 amplitude decreases [149, 150]. While behavioural results could not explicitly highlight training- or tACS-induced modulations, ERPs showed that while the amplitude of the P300 increased for the Vis-tACS group after the training, it decreased for the Vis-Sham group. Considering previous works, the results of the present study possibly suggest that training alone led to stronger automatisation of word categorisation processes [149, 150] as observed in the Vis-Sham group in the LD task, while parietal beta-tACS promoted the availability of attentional resources for WM [145]. Accordingly, both groups showed improvement in WM capacity overall as witnessed by the behavioural improvement in the forward repetition task of the digit span, but only the Vis-tACS group substantially improved in the reordering task which requires higher attentional control to support the manipulations of representations in WM [151, 152]. The specific tACS-induced improvements in WM performance is also suggested by the plastic changes testified by the growth of IBF, which can be differently modulated during WM encoding, maintenance and retrieval [136, 137]. Therefore, results suggest that while the behavioural training alone led to unspecific improvements in WM capacity possibly supported by automatisation, tACS led to additional and more specific improvements pertaining to attentional control and executive functions. Such improvements in working memory are possibly domain-general and multi-modal as they emerge both in the digit span tasks, where verbal content must be retained and manipulated, and in the lexical decision task, where visually-presented linguistic content must be retained and classified.

Importantly, specific tACS-induced improvements in reading speed were observed in the computerised reading task. Computerised reading tasks are undoubtedly more sensible to capture subtle improvements in reading performance considering the subsecond temporal resolution and the absence of an external evaluator. Indeed, standardised and computerised tests evaluated different types of reading abilities: overt (i.e. aloud) and covert (i.e. silent) reading, respectively. The selective tACS-induced improvements of covert reading, decisively converge with the range of visuoattentional and oculomotor processes that were ameliorated by targeting the parietal areas. Furthermore, it is important to note that while overt reading is essential for learning grapheme-to-phoneme mapping [70, 71], covert reading is the most ecological and used form of reading in everyday life. Consequently, improving covert reading abilities in adults with DD could have a greater impact on daily life with respect to improving overt reading. Notably, reading fluency is directly related to the functional connectivity between the auditory and visual areas during covert reading in children with DD [153]. Therefore, ameliorating visuospatial, attentional, and oculomotor processes might also result in cross-domain improvements in phonological skills.

By targeting dysfunctional oscillatory activity while the visuoattentional system is engaged, the present study introduces the first effective multi-session neuromodulation protocol for treating DD by restoring the functionality of the dorsal visual stream in a safe and tolerable way. In fact, the present findings add fundamental knowledge for the effectiveness and safety of tACS [45, 68, 154, 155]. The successful implementation of the blinding procedure ensured that participants remained uncertain about their allocation status, allowing for an accurate attribution of the effects to the intervention itself and not to the participants' expectations of their group assignment. Effective blinding is crucial for reducing potential biases and enhancing the internal validity of the findings [156]. Importantly, none of the participants reported adverse effects in response to the prolonged beta-tACS administration (12 sessions, approximately 8 h of stimulation), underlying its safety and tolerability. Nonetheless, it is important to make some considerations concerning the impact of training/remediation procedures on individuals with different subtypes of DD. Previous studies showed that training procedures tailored on the different DD subtypes and their individual cognitive profile lead to specific improvements in the impaired sub-domains [157, 158]. Therefore, it is possible that the present tACS-based protocol targeting parietal regions might benefit individuals with visuoattentional impairments more than individuals with phonological impairments.

Taken together, our findings confirm that the combined administration of beta-tACS and behavioural training has the potential to enhance perceptual and cognitive functions, resulting in faster and more robust positive clinical outcomes, compared to behavioural training alone. This integrated approach is a promising avenue not only for adults, but also for children affected by reading disorders, for whom initial evidence of safety and tolerability of electrical neuromodulation in single sessions have been recently reported [159–161]. Such advancements could lead the way for the development of more effective therapeutic interventions embracing oscillatory neuromodulation in a broad range of neurodevelopmental, neuropsychiatric and neurodegenerative disorders, thereby enhancing the overall quality of life for the many individuals with cognitive impairments.

## Supplementary Information

Supplementary Material 1.

## Data Availability

The data of this study are available from the corresponding author upon reasonable request and data sharing agreement stipulated with the ethical committee which approved the study protocol.
