# Supplementary material for: A new effective treatment for dyslexia based on dorsal visual stream neuromodulation
Source: J Neurodev Disord. 2026 Jan 31;18:11. doi: 10.1186/s11689-026-09675-3 (PMC12947359; doi:10.1186/s11689-026-09675-3)
Supplement: Supplementary file 1 — Supplementary Material 1. [file 11689_2026_9675_MOESM1_ESM.pdf]

Supplementary Materials

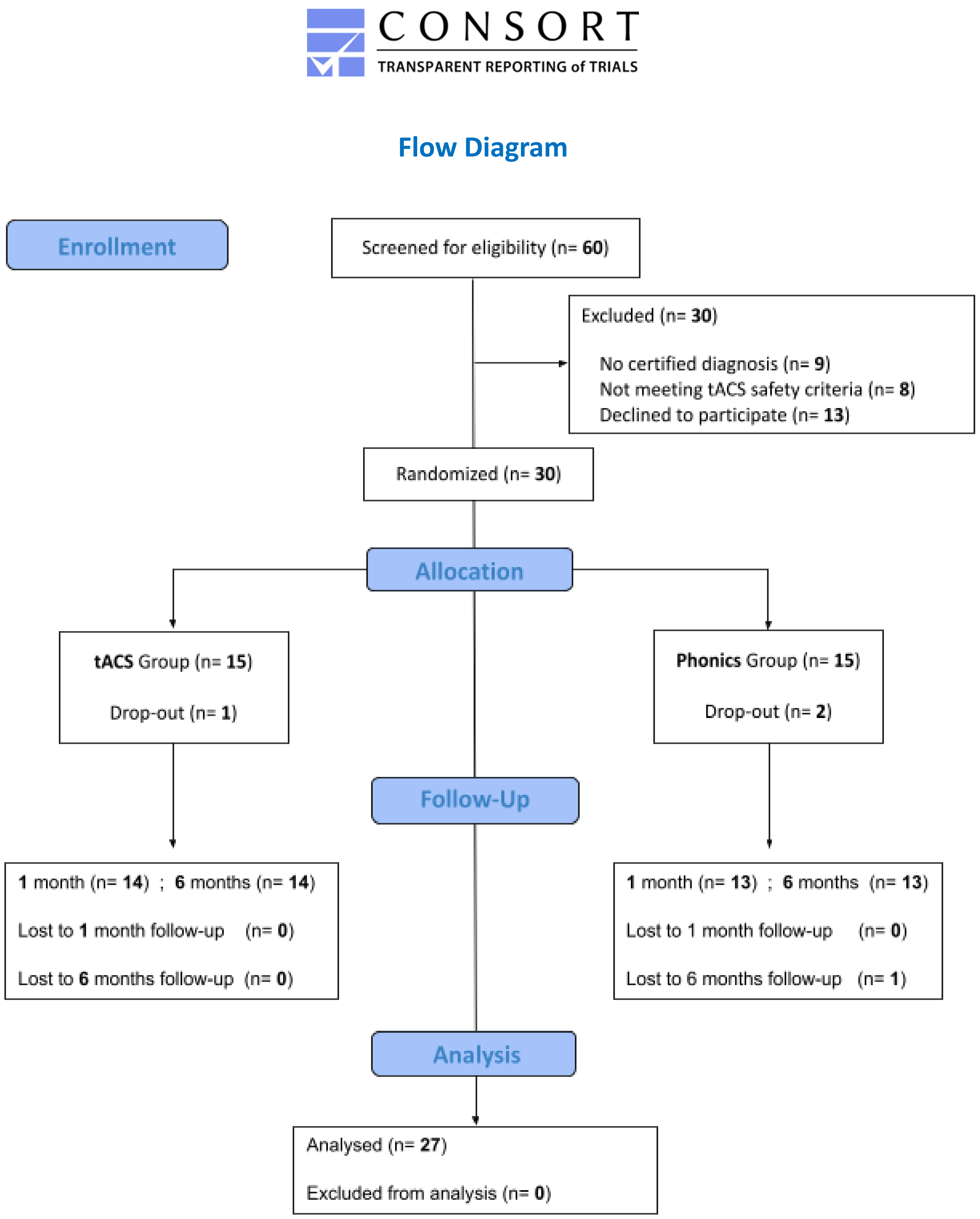

Figure S1: The Consolidated Standards of Reporting Trials (CONSORT) flowchart for the clinical trial. *The Vis-tACS group underwent a visuo-attentional reading training combined*

with 18 Hz tACS over posterior parietal cortices, the Vis-Sham group underwent the same visuo-attentional reading training combined with sham stimulation.

1. Supplementary Results

1.1. Chi-square and ANOVA tables

In the following section, the complete results of the chi-squared tests run on the mixed models of neuropsychological, behavioural, gaze, and EEG data are presented as well as the full ANOVAs run on ERP data from the Lexical Decision and the Coherent Dot Motions. At the end of the section, results pertaining to the analysis of the group allocation guess and the sensations perceived during the stimulation are reported.

1.2. Chi-square tables of neuropsychological testing (Table S1)

Text Reading - Speed

Effect	χ^2	Df	p-value
Group	0.032	1.000	0.858
Testing Session	24.481	2.000	**< .001**
Group x Testing Session	0.694	2.000	0.707

Analysis of Deviance Table (Type II Wald χ^2 test)

Text Reading - Errors

Effect	χ^2	Df	p-value
Group	0.014	1.000	0.905
Testing Session	0.656	2.000	0.72
Group x Testing Session	1.409	2.000	0.494

Analysis of Deviance Table (Type II Wald χ^2 test)

Digit Span - All subtests

Effect	χ^2	Df	p-value
Group	0.432	1.000	0.511
Testing Session	13.132	2.000	**0.001**
Group x Testing Session	4.190	2.000	0.123

Analysis of Deviance Table (Type II Wald χ^2 test)

Words Reading - Speed

Effect	χ^2	Df	p-value
Group	0.179	1.000	0.672
Testing Session	64.412	2.000	**< .001**
Group x Testing Session	0.534	2.000	0.766

Analysis of Deviance Table (Type II Wald χ^2 test)

Words Reading - Errors

Effect	χ^2	Df	p-value
Group	0.716	1.000	0.398
Testing Session	3.409	2.000	0.182
Group x Testing Session	1.375	2.000	0.503

Analysis of Deviance Table (Type II Wald χ^2 test)

Digit Span - Forward Repetition

Effect	χ^2	Df	p-value
Group	0.595	1.000	0.44
Testing Session	13.303	2.000	**0.001**
Group x Testing Session	2.902	2.000	0.234

Analysis of Deviance Table (Type II Wald χ^2 test)

Pseudowords Reading - Speed

Effect	χ^2	Df	p-value
Group	0.268	1.000	0.605
Testing Session	70.682	2.000	**< .001**
Group x Testing Session	0.239	2.000	0.888

Analysis of Deviance Table (Type II Wald χ^2 test)

Pseudowords Reading - Errors

Effect	χ^2	Df	p-value
Group	0.284	1.000	0.594
Testing Session	2.602	2.000	0.272
Group x Testing Session	1.321	2.000	0.517

Analysis of Deviance Table (Type II Wald χ^2 test)

Digit Span - Backward Repetition

Effect	χ^2	Df	p-value
Group	0.325	1.000	0.569
Testing Session	0.694	2.000	0.707
Group x Testing Session	1.131	2.000	0.568

Analysis of Deviance Table (Type II Wald χ^2 test)

RAN letter & colors naming test - Speed

Effect	χ^2	Df	p-value
Group	0.060	1.000	0.806
Testing Session	13.274	2.000	**0.001**
Group x Testing Session	3.439	2.000	0.179

Analysis of Deviance Table (Type II Wald χ^2 test)

RAN letter & colors naming test - Errors

Effect	χ^2	Df	p-value
Group	4.433	1.000	**0.035**
Testing Session	0.704	2.000	0.703
Group x Testing Session	2.514	2.000	0.285

Analysis of Deviance Table (Type II Wald χ^2 test)

Digit Span - Reordering

Effect	χ^2	Df	p-value
Group	0.102	1.000	0.749
Testing Session	6.404	2.000	**0.041**
Group x Testing Session	6.196	2.000	**0.045**

Analysis of Deviance Table (Type II Wald χ^2 test)

1.3. Chi-squared tables of computerised reading - behavioural data (Table S2)

Self-paced Reading - Letter reading time

Effect	χ^2	Df	p-value
Group	2.593	1.000	0.107
Training Session	567.955	1.000	0.000
Recording Time	136.210	1.000	0.000
Initial Reading Time	24.140	1.000	0.000
Group x Training Session	7.931	1.000	0.005
Group x Recording Time	5.131	1.000	0.023
Training Session x Recording Time	5.932	1.000	0.015
Group x Initial Reading Time	2.209	1.000	0.137
Training Session x Initial Reading Time	200.093	1.000	0.000
Recording Time x Initial Reading Time	6.642	1.000	0.010
Group x Training Session x Recording Time	5.190	1.000	0.023
Group x Training Session x Initial Reading Time	262.617	1.000	0.000
Group x Recording Time x Initial Reading Time	0.051	1.000	0.821
Training Session x Recording Time x Initial Reading Time	3.292	1.000	0.070
Group x Training Session x Recording Time x Initial Reading Time	0.162	1.000	0.687

Analysis of Deviance Table (Type II Wald χ^2 test)

Fast-paced Reading

Effect	χ^2	Df	p-value
Group	0.134	1.000	0.715
Training Session	428.396	1.000	0.000
Initial Reading Time	32.463	1.000	0.000
Group x Training Session	11.498	1.000	0.001
Group x Initial Reading Time	1.174	1.000	0.279
Training Session x Initial Reading Time	241.611	1.000	0.000
Group x Training Session x Initial Reading Time	506.201	1.000	0.000

Analysis of Deviance Table (Type II Wald χ^2 test)

1.4. Chi-squared tables of computerised reading - gaze data (Table S3)

Self-paced Reading - Regressive saccades

Effect	χ^2	Df	p-value
Recording Time	38.841	1.000	0.000
Group	4.226	1.000	0.040
Training Session	92.108	1.000	0.000
Recording Time x Group	0.584	1.000	0.445
Recording Time x Training Session	0.705	1.000	0.401
Group x Training Session	75.641	1.000	0.000
Recording Time x Group x Training Session	0.458	1.000	0.498

Analysis of Deviance Table (Type II Wald χ^2 test)

1.5. Chi-squared tables of RS-EEG (Table S4)

RS-EEG - Beta Power

Effect	χ^2	Df	p-value
Group	0.118	1.000	0.731
Recording Time	64.751	1.000	0.000
Training Session	23.951	1.000	0.000
Channel	54.135	11.000	0.000
Group x Recording Time	2.905	1.000	0.088
Group x Training Session	8.479	1.000	0.004
Recording Time x Training Session	0.548	1.000	0.459
Group x Channel	75.045	11.000	0.000
Recording Time x Channel	9.195	11.000	0.604
Training Session x Channel	5.049	11.000	0.929
Group x Recording Time x Training Session	2.834	1.000	0.092
Group x Recording Time x Channel	3.878	11.000	0.973
Group x Training Session x Channel	7.416	11.000	0.764
Recording Time x Training Session x Channel	1.370	11.000	1.000
Group x Recording Time x Training Session x Channel	3.344	11.000	0.985

Analysis of Deviance Table (Type II Wald χ^2 test)

RS-EEG - IBF

Effect	χ^2	Df	p-value
Group	0.212	1.000	0.645
Recording Time	1.689	1.000	0.194
Training Session	11.326	1.000	0.001
Channel	81.128	11.000	0.000
Group x Recording Time	5.599	1.000	0.018
Group x Training Session	10.340	1.000	0.001
Recording Time x Training Session	0.259	1.000	0.611
Group x Channel	72.568	11.000	0.000
Recording Time x Channel	3.644	11.000	0.979
Training Session x Channel	7.016	11.000	0.798
Group x Recording Time x Training Session	0.066	1.000	0.798
Group x Recording Time x Channel	8.996	11.000	0.622
Group x Training Session x Channel	5.081	11.000	0.927
Recording Time x Training Session x Channel	5.339	11.000	0.914
Group x Recording Time x Training Session x Channel	4.233	11.000	0.963

Analysis of Deviance Table (Type II Wald χ^2 test)

1.6. Chi-squared tables of Sensations and Allocation Guess (Table S6, S7)

After each tACS session, participants filled out a questionnaire to evaluate their sensations during the stimulation (Fertonani et al., 2015). They could report different kinds of sensations (burning, fatigue, pain, itching, heat, pinch, metallic aftertaste or other) and indicate the respective magnitude on a 4-points scale (0 = None; 1 = Mild; 2 = Moderate; 3 = Considerable; 4 = Strong). In the following table (Table S6) the proportion of reported sensations for each session is reported for the Active group (Vis-tACS) and the Passive group (Vis-Sham). In the subsequent table (Table S7), the p-values (FDR-corrected) of the chi-squared test which checked whether the proportion of participants who reported sensations were different between the Active and the Passive group. NA values refer to tests in which the proportions were 0 for both groups (see Table S6). After the whole training participants were asked whether they thought they received an active or placebo/sham stimulation. A chi-squared chi-square test showed that $\chi^2(1) = 1.30$, $p = .25$ showed that responses were equally distributed across stimulation protocols meaning that participants' guesses were not differentiated by group.

1.7. Chi-square table of sensations (Table S6)

		Session											
**Group**	**Sensation**	1	2	3	4	5	6	7	8	9	10	11	12
Active (Vis-tACS) N = 14	Burning	0.33	0.4	0.27	0.33	0.27	0.27	0.53	0.27	0.2	0.2	0.07	0.33
	Fatigue	0.4	0.27	0.33	0.33	0.27	0.33	0.27	0.27	0.2	0.27	0.13	0.27
	Heat	0.33	0.27	0.13	0.13	0.27	0.13	0.2	0.07	0.07	0.13	0.07	0.2
	Itch	0.53	0.53	0.4	0.47	0.67	0.47	0.53	0.4	0.4	0.27	0.33	0.53
	Metal	0.07	0	0	0	0	0	0	0	0	0	0	0
	Other	0.2	0.33	0.27	0.13	0.07	0.13	0.07	0	0	0.07	0	0
	Pain	0	0	0.2	0.2	0.2	0.27	0.13	0	0.07	0	0.07	0.2
	Pinch	0.07	0.13	0	0	0.13	0.07	0.13	0.07	0	0	0	0
Passive (Vis-Sham) N = 13	Burning	0.07	0.13	0.33	0.13	0.13	0.07	0.13	0.07	0.07	0.27	0.13	0
	Fatigue	0.13	0.2	0.07	0.2	0.13	0.07	0.13	0.07	0.13	0.07	0.13	0.07
	Heat	0.07	0.13	0.27	0.07	0.13	0.07	0.07	0.07	0.2	0.2	0.07	0.07
	Itch	0.4	0.33	0.33	0.47	0.47	0.2	0.33	0.2	0.33	0.4	0.4	0.4
	Metal	0	0.07	0	0	0	0	0	0	0	0	0.07	0
	Other	0.13	0.2	0.07	0.07	0.07	0.07	0	0.13	0	0.13	0.07	0
	Pain	0.13	0	0.07	0.07	0	0	0	0	0.07	0.07	0.07	0
	Pinch	0.07	0	0.13	0.07	0.07	0	0.07	0	0	0	0.07	0

1.8. Chi-square table of allocation guess (Table S7)

		Session											
**Test**	**Sensation**	**1**	**2**	**3**	**4**	**5**	**6**	**7**	**8**	**9**	**10**	**11**	**12**
Active vs Passive	Burning	0.66	0.66	1	0.67	0.87	0.66	0.3	0.66	0.87	1	1	0.3
	Fatigue	0.66	1	0.66	0.91	0.91	0.66	0.91	0.66	1	0.66	1	0.66
	Heat	1	1	1	1	1	1	1	1	1	1	1	1
	Itch	1	1	1	1	1	1	1	1	1	1	1	1
	Metal	1	1	NA	NA	NA	NA	NA	NA	NA	NA	1	NA
	Other	1	1	1	1	1	1	1	1	NA	1	1	NA
	Pain	0.84	NA	0.84	0.84	0.73	0.73	0.84	NA	1	1	1	0.73
	Pinch	1	1	1	1	1	1	1	1	NA	NA	1	NA
